# SBA-16 Cage-Like Porous Material Modified with APTES as an Adsorbent for Pb^2+^ Ions Removal from Aqueous Solution

**DOI:** 10.3390/ma13040927

**Published:** 2020-02-19

**Authors:** Viviana Palos-Barba, Abigail Moreno-Martell, Verónica Hernández-Morales, Carmen L. Peza-Ledesma, Eric M. Rivera-Muñoz, Rufino Nava, Barbara Pawelec

**Affiliations:** 1 División de Investigación y Posgrado, Facultad de Ingeniería, Universidad Autónoma de Querétaro, Centro Universitario, 76010 Querétaro, Mexico; vvplbr@gmail.com (V.P.-B.); morenomartell@gmail.com (A.M.-M.); 2 Centro de Física Aplicada y Tecnología Avanzada, Universidad Nacional Autónoma de México, Departamento de Nanotecnología, A.P. 1-1010 Querétaro, Mexico; vero_hm@hotmail.com (V.H.-M.); carmenpez@gmail.com (C.L.P.-L.); emrivera@fata.unam.mx (E.M.R.-M.); 3 Instituto de Catálisis y Petroleoquímica, CSIC, Cantoblanco, 28049 Madrid, Spain; bgarcia@icp.csic.es

**Keywords:** NH_2_-SBA-16, mesoporous silica, Pb(II) removal, adsorption

## Abstract

Tridimensional cubic mesoporous silica, SBA-16, functionalized with aminopropyl groups, were employed as adsorbents for Pb^2+^ ion removal from aqueous solution. The adsorption capacity was investigated for the effect of pH, contact time, temperature, and concentration of 3-aminopropyltriethoxysilane (APTES) employed for adsorbent functionalization. The textural properties and morphology of the adsorbents were evaluated by N_2_ physisorption, small-angle X-ray diffraction (XRD), diffuse reflectance spectroscopy (UV-vis), and transmission electron microscopy (TEM). The functionalization of the SBA-16 was evaluated by elemental analysis (N), thermogravimetric analysis (TG), Fourier transform infrared spectroscopy (FT-IR), and X-ray photoelectron spectroscopy (XPS). Batch adsorption studies show that the total Pb^2+^ ions removal was archived on adsorbent having an optimized amount of aminopropyl groups (2N-SBA-16). The maximum of Pb^2+^ ions removal occurred at optimized adsorption conditions: pH = 5–6, contact time 40 min, and at a low initial lead concentration in solution (200 mg L^−1^). Under the same adsorption conditions, the amino-functionalized SBA-16 with cubic 3D unit cell structure exhibited higher adsorption capability than its SBA-15 counterpart with uniform mesoporous channels.

## 1. Introduction

Recently, there is an increased interest in the removal of toxic heavy metals from wastewaters using adsorption technology and low-cost lignocellulosic materials as adsorbents [1,2,3,4,5,6]. However, the natural adsorbents, such as wheat straw, coconut coir pith, tree fern, etc., demonstrated a low mechanic resistance to abrasive forces, low loading capacities, and relatively weak interactions with heavy metal cations [7,8,9]. To overcome these drawbacks, recent studies are focused on the use as adsorbents the mesoporous silica materials, such as MCM-41 [10,11], SBA-15 [12,13,14,15,16,17,18,19,20,21,22,23,24,25,26,27,28,29,30] and SBA-16 [30,31,32,33,34,35,36] due to their ordered mesoporous structure with high surface area and appropriate pore size for the accommodation of guest molecules [37]. Notwithstanding, as the pure siliceous materials have only silanol groups on their surface, it is necessary to create specific binding sites by functionalization of the adsorbent surface with chemically bonded groups, such as -SH [15,16,17], -COOH [14,17], and -NH_2_ [4,10,12,19,20,21,22,23,24]. In particular, the effect of amine functionalization of SBA-15 and SBA-16 has been extensively studied for removal of metal ions from various sources [12,19,20,21,22,23,24,27,28,29,30,31,32,33,34,35,36], or controlled drug release [25,26,37] because of easy accommodation of the aminopropyl groups in the ordered pore structure of SBA-15 adsorbent and the efficient van der Waals electrostatic interaction of amino groups with Pb^2+^ upon optimized adsorption conditions, as it was confirmed by us previously [12].

In order to improve the efficiency of the adsorption process, we investigated the adsorption capacity of SBA-16 substrate, scarcely employed for heavy ions removal [31,32,33,34,35,36]. The SBA-16 substrate exhibits an interesting three-dimensional cubic-like (*Im3m*) structure with interconnected micro-and mesopores, large surface area, and adequate pore diameter for the accommodation of the guest molecules [38]. Recently, the potential application of SBA-16 material synthesized from rice husk ash as an adsorbent for acetone vapors was investigated by Zeng and Bai [32]. It was found that SBA-16 exhibited superior adsorption capacity than ZSM-5 and MCM-41. This was ascribed to their higher specific surface area, which provides more adsorption sites for adsorption of acetone vapors, and the presence of micropores [32]. Lesaint et al. [35] used SBA-15- and SBA-16 mesoporous silica functionalized with mercaptopropyl groups as adsorbents for Hg^2+^ ions removal from aqueous solution. For both types of adsorbents, the evaluation of the diffusion rates of Hg^2+^ species in the mesoporous solids showed faster binding rates for the adsorbents functionalized by post-synthesis grafting than for their counterparts prepared by the one-step co-condensation route [35]. β-diketone functionalized SBA-15 and SBA-16 mesoporous materials were successfully used for the removal of copper from aqueous solution by Ouargli et al. [27]. Their SBA-16-based adsorbent exhibited a larger copper extraction capacity than its SBA-15-based counterpart. 

This work aims to study the adsorption capacity of SBA-16 functionalized with variable amounts of amine groups. Considering that lead is one of the most toxic pollutants released into aquatic systems from many industrial processes [12], the adsorption capacity of amine-functionalized SBA-16 adsorbents was investigated for the removal of Pb^2+^ ions from aqueous solution. In order to optimize adsorption conditions, the adsorption experiments were conducted at varying adsorbent and ligand ratio (TEOS/APTES ratio), contact time, pH, initial Pb^2+^ concentration, and temperature. The physicochemical characteristics of the amine-modified SBA-16 adsorbents before and after Pb^2+^ adsorption were investigated by elemental analysis, thermal analysis, N_2_ adsorption-desorption isotherms, Fourier-transform infrared spectroscopy, DRS UV-vis, and XPS techniques. The influence of the adsorbent morphology on the adsorbent capacity is discussed.

## 2. Materials and Methods 

### 2.1. Preparation of the Pure SBA-16

The SBA-16 substrate was synthesized according to the method described previously [22,23]. In the synthesis, 8 g of the triblock Pluronic F127 copolymer (EO_106_PO_70_EO_106_, Sigma-Aldrich, 99%) was dissolved in a solution of 60 mL of deionized water and 240 mL of 2M HCl. After 1 h of stirring, 26 mL of tetraethyl orthosilicate (TEOS, Aldrich, 98%) was added to the water-copolymer solution. This mixture was further stirred for 24 h at a constant temperature of 35 °C. The obtained suspension was transferred into a tightly closed polypropylene bottle and kept at 80 °C for 24 h without stirring. The precipitated solid was filtered and washed thoroughly with deionized water. After drying in air at 110 °C, the solid was calcined at 500 °C for 6 h to remove the organic template. 

### 2.2. Functionalization of the SBA-16 with APTES

The SBA-16 mesoporous silica functionalized with 3-aminopropyltriethoxysilane (APTES, Aldrich, 97%) was prepared according to the procedure described in an earlier contribution [1]. Briefly, 1.0 g of dry SBA-16 was introduced into APTES-ethanol solution containing different amounts of APTES while continuously stirring. The molar ratio of TEOS to APTES was 1:0.2, 1:0.3 and 1:0.4, hereinafter called 2.6N/SBA-16, 3.8N/SBA-16, and 5.1N/SBA-16. Then, the liquid suspension was stirred at room temperature in an inert atmosphere for 30 min, whereas deionized water was slowly added to conduct the hydrolysis process of the organic functional group alkoxide from APTES. Then, the solid was dried overnight at room temperature followed by heating at 110 °C for 24 h. The SBA-16 mesoporous materials modified with amine groups will be referred to hereafter as xN/SBA-16, where x corresponds to the N content in the amine-functionalized adsorbents.

### 2.3. Characterization Techniques

Quantitative determination of the nitrogen content was measured by elemental microanalysis on a elementar Analysensysteme GmbH-vario EL III Element Analyzer (Langenselbold, Germany). The textural properties of the adsorbents were evaluated using a Micromeritics TriStar 3000 apparatus (Micromeritics, Norcross, GA, USA) from the nitrogen adsorption-desorption isotherms recorded at −196 °C. Before the measurement, the samples were degassed at 150 °C for 24 h under a vacuum (10^−4^ mbar). Their specific total surface area was calculated using the Brunauer-Emmett-Teller (BET) method [24]. The pore size distribution was calculated from the adsorption branch of the N_2_ isotherm using the BJH method. The total pore volume (V_total_) was calculated from the amount of nitrogen adsorbed at a relative pressure of 0.99 [25].

X-ray diffraction (XRD) patterns of the powder samples were carried out using the Cu Kα radiation with a wavelength of 1.5406 Å in the range: 0.5–3 (low-angle) on a PANalytical diffractometer (Almelo, The Netherlands). The unit cell parameter (a_o_) was calculated using Equation (1):

a_o_ = d_110_√2 (1)

where d_110_ is the position of the (110) diffraction line (from low-angle XRD). The pore-wall thickness (*w*_t_), which corresponds to the distance between the centers of adjacent mesopores, was estimated using the Equation (2) [26]:

*w*_t_ = (a_o_√3/2) − *d*_p_ (2)

where *d*_p_ is the mean pore diameter. 

Fourier transform-IR (FT-IR) spectra of the framework vibration (400–1800 cm−1 range) were recorded on a JASCO FT/IR-6300 spectrophotometer (JASCO, Easton, WA, USA) using the potassium bromide pellet method. The stability of the aminopropyl groups was determined by thermogravimetric analysis (Model TGA 2950, TA Inc, New Castle, USA) by weight loss of the samples during their heating in an atmosphere of nitrogen (a heating rate of 5 °C·min^−1^). The materials after lead adsorption were analyzed by UV-vis diffuse reflectance spectra at room temperature on a CARY 5000 UV-Vis-NIR VARIAN instrument (Varian, Santa Clara, CA, USA). X-ray photoelectron spectroscopic studies of the adsorbents were recorded on a VG Escalab 200R spectrometer (Vacuum Generators, Crowborough, UK) equipped with a hemispherical electron analyzer (Vacuum Generators, Crowborough, UK), using an MgKα (hν = 1253.6 eV, 1 eV = 1.603 × 10^−19^ J) X-ray source. The details of the XPS measurements are reported elsewhere [1].

### 2.4. Adsorption Experiments

The Pb^2+^ adsorption on the SBA-16 and xN/SBA-16 adsorbents was carried out in a batch reactor (Facultad de Ingeniería, Universidad Autónoma de Querétaro, Querétaro, Mexico) using deionized water in all experiments. The various parameters investigated for Pb^2+^ adsorption were: contact time (20–120 min, with ranges of 20 min), reaction temperature (30, 35, and 40 °C), and pH of solution (the pH value was adjusted using HNO_3_ or NaOH 0.1 M). Lead solutions were obtained from lead nitrate (Baker, 99.92%). In a typical run, 0.1 g of adsorbent and 20 mL of 200 mg L^−1^ of lead solution were placed in a shaker (Thermo Scientific, Waltham, MA, USA) at 150 rpm for 1 h (pH = 5) at room temperature. After this period of stirring, the suspension was centrifuged at 2500 rpm for 5 min, and finally, the adsorbents were recovered from through filtration. Inductively coupled plasma atomic emission spectroscopy (ICP-AES) was employed to determine the initial and final lead concentrations with a Perkin Elmer Optima 3300 DV spectrometer (Perkin Elmer, Waltham, MA, USA) calibrated with 0–10 mg L^−1^ stock solutions. The emission line used was in accordance with the Environmental Protection Agency (EPA) standard method [27]. For each equilibrium point, the amount of adsorbed Pb^2+^ was determined by the difference between initial and final metal concentrations in the solution. Replica experiments indicated that associated error was within ± 3%. The percentage of adsorbed Pb^2+^ was calculated according to Equation (3):

% Pb(II) adsorbed = [(C*_i_* − C*_f_*)/C*_i_*] × 100% (3)

where C*_i_* and C*_f_* are the initial and final Pb(II) ions concentration in the solution, respectively. 

The adsorption capacity of Pb^2+^ per unit weight of the modified adsorbent at time *t*, (Q*_t_*; mg g^−1^), was calculated from the mass balance in Equation (4):

Q*_t_* = (C*_i_* − C*_f_*) × V/m (4)

where C*_i_* (mg L^−1^) and C*_f_* (mg L^−1^) are the initial and final concentrations of Pb^2+^ at time *t*, respectively, V is the volume of the aqueous solution, and m is the mass of adsorbent.

## 3. Results and Discussion

### 3.1. Chemical Analysis and Low-Angle X-ray Diffractions

The nitrogen content of the amine-functionalized adsorbates is listed in Table 1. As expected, the nitrogen content increases with an increase of TEOS/APTES ratio. The SBA-16 substrate exhibits interesting morphology consisting of the three-dimensional channel systems corresponding to *Im3m* space group symmetry and uniform cage-like mesopores with a cubic symmetry [22]. Its 3D structure consisting of ordered and interconnected spherical mesopores makes it easily accessible for Pb(II) ions. The reaction of silanol groups of calcined SBA-16 substrate with ethoxysilanes resulted in the anchorage of aminopropyl functional groups. 

**Figure 1:**
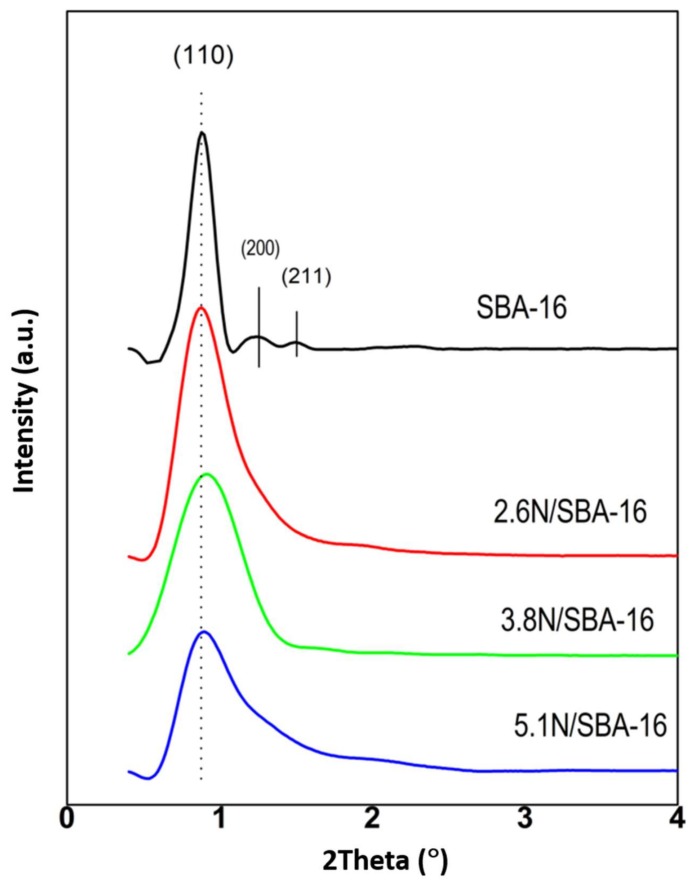
Small-angle X-ray diffraction patterns of pure SBA-16 and amine-functionalized SBA-16 adsorbents.

The possible structure changes after SBA-16 grafting with -NH_2_ groups was investigated by powder low-angle XRD technique (Figure 1). The XRD pattern of the calcined SBA-16 shows reflections at 2*θ* = 0.88°, 1.22°, and 1.50° indexed as [110], [200], and [211]. All these peaks are associated with the cubic symmetry (*Im3m*) of cage-structured mesoporous SBA-16 silica material. Contrary to the bare SBA-16 substrate, the low-angle XRD patterns of the *x*N/SBA-16 samples did not show higher Miller indices reflections ([200] and [211] for characteristic cubic *Im3m* spatial groups) suggesting that functionalization of SBA-16 substrate leads to a structure with minor pore arrangement and a major presence of nitrogen molecules [28]. 

**Figure 2:**
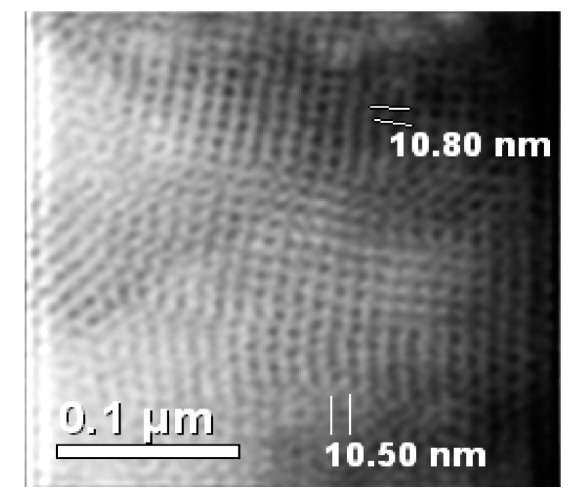
Transmission electron microscopy (TEM) image of the 3.8N/SBA-16 adsorbent showing a well-ordered cubic array of unit cells.

However, a transmission electron micrograph (TEM) image of the representative 3.8N/SBA-16 sample showed a well-ordered *Im3m* cubic array of mesopores (Figure 2). Therefore, it can be inferred that the mesoporous structure of SBA-16 adsorbent did not suffer significant modification after its functionalization with amine groups. For 5.1N/SBA-16, the low intensity of its peak at reflection [110] is linked with a high wall thickness/pore size ratio (Table 1) that originated from a large number of micropores located near the wall surface the pore walls [29].

To determine both structure and symmetry of the synthesized samples, the cubic unit cell parameters and the wall thickness were calculated using XRD data and Equations (1) and (2), respectively (Table 2). The cubic unit cell parameter (*a*_0_) of all samples was found to be in the range 13.91–14.19 nm, confirming their *Im3m* structure [30]. Both 2.6N/SBA-16 and 3.8N/SBA-16 samples show smaller cubic unit cell parameters than the bare SBA-16 sample indicating the possible location of amine groups within the porous structure of the SBA-16 substrate. The higher thermal and hydrothermal stability with respect to the bare SBA-16 substrate could be inferred [31]. Noticeably, the functionalization of SBA-16 material with an optimized amount of amine groups did not change the wall thickness (3.8N/SBA-16). On the contrary, an increase of the pore wall thickness observed for the 5.1N/SBA-16 is probably due to the location of the amine group within the pores in addition to the external surface, in good agreement with a large decrease of the specific surface area calculated using Brunauer-Emmett-Teller (BET) equation.

### 3.2. Textural Properties

**Figure 3:**
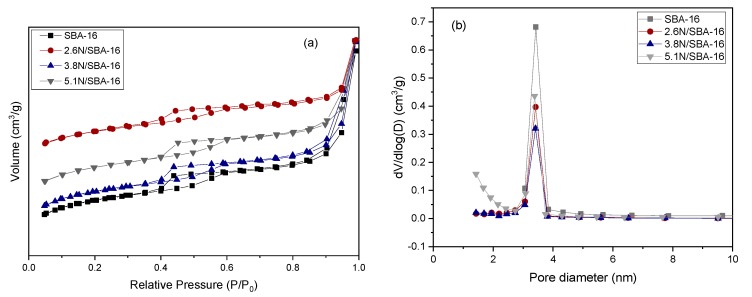
Non-functionalized SBA-16 solid and amino-functionalized adsorbents: (**a**) N_2_ adsorption-desorption isotherms, and (**b**) Pore size distribution.

Further insight into the textural properties of the samples was evaluated by N_2_ adsorption-desorption isotherms. To illustrate this, Figure 3a shows the N_2_ isotherms of bare SBA-16, and amino-functionalized materials. Irreversible type IV isotherms with type an H2 hysteresis loop was observed in all amino-functionalized and bare SBA-16 material. Type H2 hysteresis loop in the range from 0.4 to 0.6 P/P_0_ is distinctive from materials with pores with diameters between 3–6 nm [32,33], a structural porosity that can be detected because of the characteristic networking pore model from the SBA-16 material. The hysteresis loop observed at high relative pressures corresponds to the textural porosity due to the voids formed between particles during the analysis. These results are typical of materials with cubic pores and pore network connectivity like SBA-16 and reveal that the mesoporous nature of the material is preserved even though the grafting has occurred, as shown in samples 2.6N/SBA-16, 3.8N/SBA-16 and 5.1N/SBA-16. At approximately P/P0 ≈ 0.4, an explicit change is present, and it is related to capillary condensation that fills the mesopores. When the substrate is functionalized with (-NH_2_), there is an increase of the volume of adsorbed nitrogen, and the inflection point of the step shifted only slightly from relative pressure of 0.4 to 0.41. The minimum value of adsorbed nitrogen suggests the modification in the pores with amine groups, while the slight relative pressure shift of the step is indicative of mesopore sizes, a key requirement to be an efficient adsorbent.

The pore size distributions of bare SBA-16 and *x*N/SBA-16 substrates are displayed in Figure 3b. Bare SBA-16 and *x*N/SBA-16 samples showed a uniform and narrow pore size distributions centered at about 3.4 nm, demonstrating that the incorporation of APTES to the SBA-16 does not alter the cubic structure of the raw SBA-16 substrate. Table 1 compiles the values of some textural parameters (BET area, mesopore volume, and pore diameter) for the bare SBA-16 sample and their NH_2_-modified counterparts. As expected, the BET area and the mesoporous volume strongly decreased after modification according to the sequence SBA-16 (650 m^2^/g) >> 2.6N/SBA-16 (507 m^2^/g) > 3.8N/SBA-16 (500 m^2^/g) > 5.1N/SBA-16 (494 m^2^/g), suggesting that the grafted species appear concentrated not only on the external surface but also within the mesopore network of the SBA-16 substrate. Interestingly, regardless of APTES concentration, all amine-modified samples exhibit very similar specific surface area (in the range 494–507 m^2^/g). It is known that the SBA-16 possesses micropores located within the walls of primary mesopores forming a three-dimensional channel system with a connection between the mesopores [34]. The micropores are formed during the synthesis of SBA-16 due to the penetration of more hydrophilic EO chains of the tri-block copolymer in the silica wall [35,36]. Thus, the large loss of total pore volume after SBA-16 modification with APTES should also be explained in terms of blocking micropores by large ligand groups.

### 3.3. FTIR of Framework Vibration

**Figure 4:**
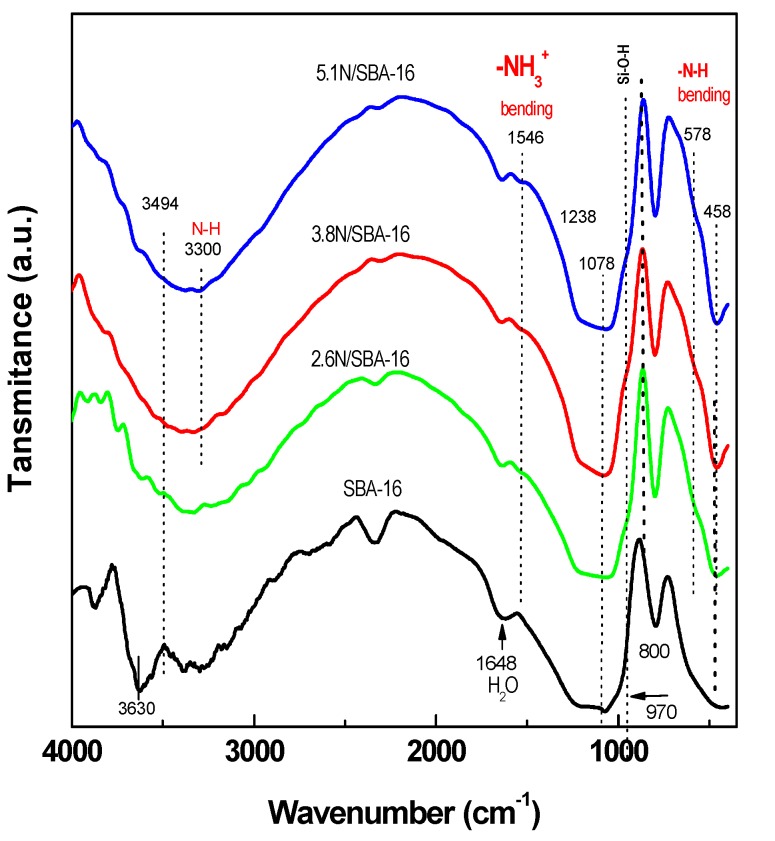
Fourier transform infrared spectra of the framework vibration region of the SBA-16 substrate before and after its grafting with aminopropyl groups.

Direct probe on the functionalization of SBA-16 surface by -NH_2_ groups were obtained from the FTIR spectra of framework vibrations of *x*N/SBA-16 samples. In Figure 4 are compared the FTIR-KBr spectra of the 2.6N/SBA-16, 3.8N/SBA-16 and 5.1N/SBA-16 samples with those of bare SBA-16 material used as a reference. The spectra show the absorption bands assigned to the structure sensitive vibrations between (SiO_4_) tetrahedra at about 800 and 1078 cm^−1^, as well as the structure insensitive vibrations assigned to the (SiO_4_) tetrahedra at about 458 and 1238 cm^−1^ [37]. For the pure SBA-16, the band at 1078 cm^−1^ exhibits a shoulder at about 970 cm^−1^, which is due to the Si-O-H stretching vibration of Si-O-R^+^ groups [38]. For the *x*N/SBA-16, the absence of this shoulder should be taken as indirect evidence of the functionalization of the SBA-16 substrate with amine groups. The direct evidence of the N-H bending mode vibration should be obtained from the peak at about 1646 cm^−1^ as well as from a shoulder at about 578 cm^−1^. Finally, the peak at about 1546 cm^−1^, could be ascribed to the symmetrical–NH_3_^+^ bending vibration.

More information should be obtained by analyzing a very broad band in the wavenumber range 3000–4000 cm^−1^. For the *x*N/SBA-16 samples, the N−H stretching vibration is expected to occur at about 3300 cm^−1^ [39]. Unfortunately, in the same region, all the samples exhibit bands due to silanol groups (ca. 3750 cm^−1^) and adsorbed molecular or hydrogen-bonded molecules (ca. 3630 cm^−1^) [40,41]. As seen in Figure 4, for the amine-modified samples, the band in this region is much broader than for the bare SBA-16. The widening of this band should be originated by the symmetric stretching vibration of N−H groups in the terminal amine groups cross-linked with the -SiOH groups [41].

### 3.4. Thermogravimetry (TG)

**Figure 5:**
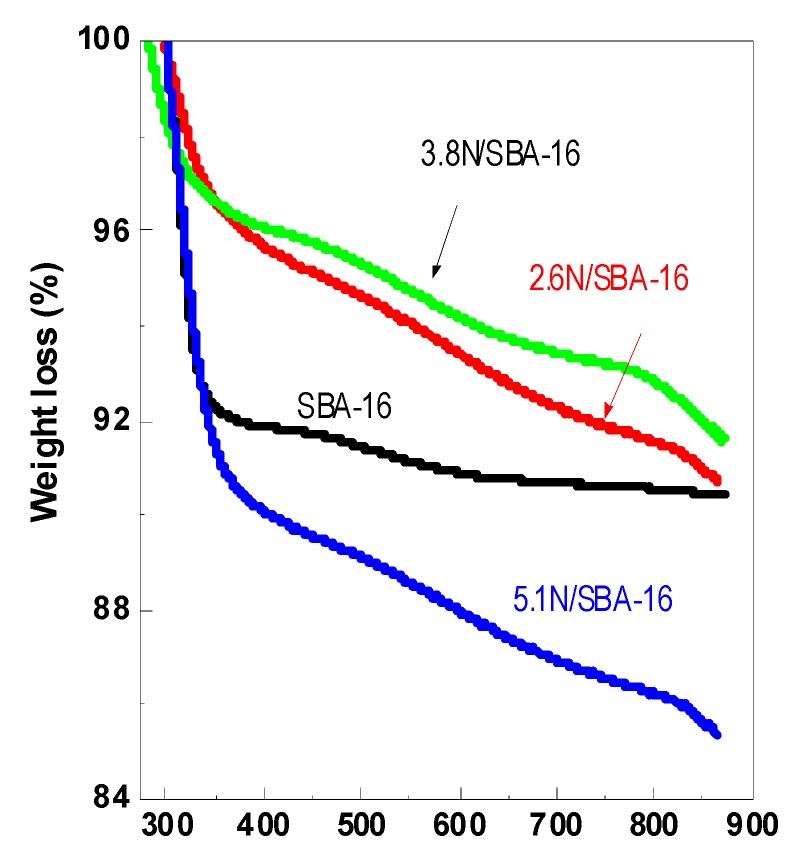
Thermogravimetric curves of SBA-16, 2.6N/SBA-16, 3.8N/SBA-16, and 5.1N/SBA-16 adsorbents.

Figure 5 displays the weight loss of the samples during thermal treatment of the adsorbents in an atmosphere of nitrogen, as determined by thermogravimetry. The thermogravimetric profiles indicate a significant weight loss, which occurs just at very low temperatures. The total weight loss is approximately 10.5wt.% from room temperature to 600 °C. It shows a first inflection point at 45 °C corresponding to dehydration of the SBA-16 material. A major weight loss, at about 250 °C, can be attributed to surface dihydroxylation or structural rearrangement of the aminopropyl groups [42]. Considering that pure SBA-16 material did not exhibit the weight loss above 300 °C, the weight loss observed in the temperature range 300–600 °C is due to the decomposition of aminopropyl groups of the *x*N/SBA-16 solids [42]. The thermogravimetric analysis corroborates the successful modification of the SBA-16 with APTES. In good agreement with the literature [42], the aminopropyl groups start to decompose at a temperature of 27 °C, higher than the boiling point of APTES liquid, indicating the chemical bonding of 3-aminopropylsilane with –OH groups of the SBA-16 material.

### 3.5. DRS UV-Vis

**Figure 6:**
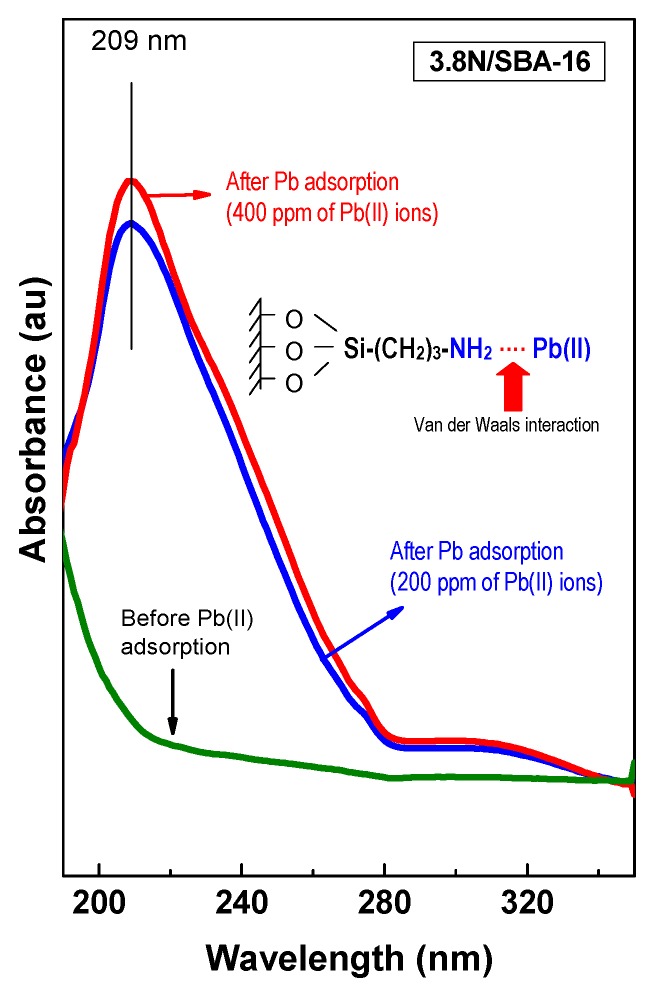
UV-vis spectra for the 3.8N/SBA-16 adsorbent after adsorption (initial Pb^2+^ concentrations in aqueous solution: 200 and 400 mg L^−1^). The scheme of the proposed van der Walls interaction of Pb^2+^ with amino group [1] is shown in the inlet of this figure.

Next to the lead adsorption with initial Pb^2+^ concentrations of 200 and 400 ppm, UV-Vis spectra were recorded to exhibit the changes on the 3.8N/SBA-16 material. Figure 6 shows only a little larger lead absorption occurring at higher Pb^2+^ ion concentration in solution (400 mg L^−1^). The scheme of Pb^2+^ ions complexation with the amino group of 3.8N/SBA-16 is shown in the inlet of Figure 6. It is assumed that van der Waals electrostatic interaction is taken place at the amino groups in the surface of the adsorbent with the Pb^2+^ ions, explaining its mechanism of adsorption [1]. The absorption spectra of all adsorbents display two bands: an intense band appears at 210 nm, and a less intense band is observed at ca. 310 nm [43]. These transitions contain both ligand-to-metal charge transfer (N 2p → Pb 6sp) and intraatomic (Pb 6s^2^ → Pb 6sp) character (for Pb in O_h_: a*_1g_^2^ → a*_1g_^1^t*_1u_^1^) [44]. Both absorption bands can be used to gain qualitative information about the affinity of Pb^2+^ for the amine groups of the *x*N/SBA-16 adsorbents.

### 3.6. X-ray Photoelectron Spectroscopy

**Figure 7:**
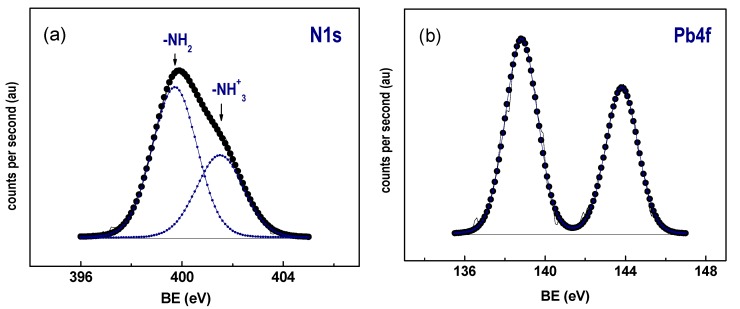
Core level spectra of 3.8N/SBA-16 adsorbate after Pb2+ adsorption: (**a**) N 1s, and (**b**) Pb4f (0.1 g of adsorbent, 20 mL of 200 mg L^−1^ aqueous Pb^2+^ solution, pH = 5.0, contact time of 60 min, and T = 30 °C).

To clarify the type of Pb species formed on the surface of the amine-functionalized SBA-16 after Pb^2+^ ion adsorption, the most optimized 3.8N/SBA-16 sample was also studied by the XPS technique. Table 3 lists the binding energies of Si 2p, O 1s, N 1s and Pb 4f_7/2_ core electrons of 3.8N/SBA-16 sample, whereas Figure 7a,b shows its N 1s and Pb 4f_7/2_ core-level spectra, respectively. For comparison purposes, some XPS data of the SBA-15-based counterpart [1] are included in Table 3. In good agreement with our previous study [1], the 3.8N/SBA-16 sample shows the O 1s peak at about 532.9 eV which is characteristic of oxygen in Si–O–Si bonds together with two peaks (Pb4f_7/2_ and Pb4f_5/2_) derived from spin-orbit splitting (Figure 7b). The binding energy of the Pb 4f_7/2_ core level appeared at 139.0 eV and 139.2 eV. According to the literature [45], the Pb 4f binding energies are stronger than the energies corresponding to the orthorhombic PbO compound (137.4 eV), and it is similar to those reported for Pb(NO_3_)_2_ (138.6 eV). There is no evidence that implies precipitation of Pb occurs as hydroxides or carbonates throughout the adsorption process with the XPS results. Finally, -NH_2_ bonds are attributed to the binding energy at 400.1 eV, while positively charged -NH_3_^+^ groups are identified at binding energies of 402.0 eV [46]. Thus, both -NH_2_ and protonated NH_3_^+^ species seem to be present previous to and afterward Pb^2+^ adsorption. -NH_2_ groups are predominant in the material, and these can be coordinated with Pb^2+^ ions through the pair of free electrons. Noticeably, the nitrogen atoms exposed in the surface for the 3.8N/SBA-16 was much lower than for its SBA-15-based counterpart prepared with the same TEOS/APTES molar ratio of 3.3 (N/Si ratio of 0.071 vs. 0.109). Besides this, both adsorbents exhibited a similar Pb/N atomic ratio (0.133 vs. 0.139), suggesting a similar surface exposure of the Pb^2+^.

### 3.7. Adsorption Experiments

**Figure 8:**
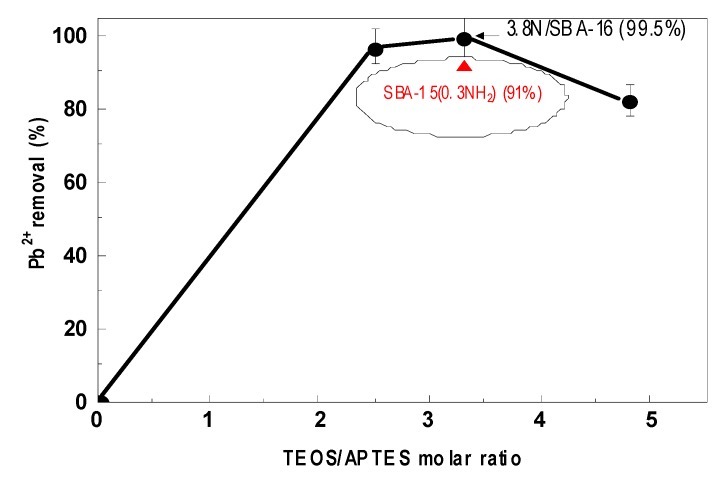
Influence of adsorbent/ligand molar ratio on the Pb^2+^ adsorption onto SBA-16-based adsorbents. For comparison purposes, the SBA-15-based adsorbent prepared with tetraethyl orthosilicate/3-aminopropyltriethoxysilane (TEOS/APTES) molar ratio of 3.3 is included (from [1]). Conditions were: 20 mL of 200 mg L^−1^ aqueous Pb^2+^ solution, 0.10 g of adsorbent, pH = 5.0, contact time of 60 min, and T = 30 °C.

Figure 8 shows the percentage of Pb^2+^ ions adsorption onto *x*N/SBA-16 adsorbents functionalized with variable amounts of aminopropyl groups and pure silica SBA-16. The adsorption was performed at pH = 5.0, temperature 30 °C, and contact time of 60 min. As seen in this Figure, the adsorption of Pb^2+^ did not occur on the pure silica SBA-16. On the contrary, all amine-functionalized SBA-16 materials were good adsorbents for Pb^2+^ removal. The adsorption of Pb^2+^ increased with an increase of the TEOS/APTES molar ratio up to 3.3. At this point, the maximum percentage of the removal of the Pb^2+^ ions from the solution was about 99% (3.8N/SBA-16). At higher -NH_2_ concentration, a drop in the Pb^2+^ removal percentage occurs (5.1N/SBA-16). Thus, in good agreement with the previous study on the SBA-15-based adsorbents [1], the best results were obtained using a TEOS/APTES molar ratio 3.3. This is probably because a large amount of APTES molecules with aminopropyl chain shielding close to silanol groups limits the entrance of Pb^2+^ ions into the inner porous structure of the SBA-16 substrate [47]. However, considering the nitrogen content determined by elemental microanalysis (Table 1), the Pb/N atomic ratio follows the trend: 2.6N/SBA-16 > 3.8N/SBA-16 > 5.1N/SBA-16. Taking into account that the same trend follows their specific surface area, one might conclude that both functionalization and textural properties of the adsorbent are important factors influencing the adsorption efficiency.

**Figure 9:**
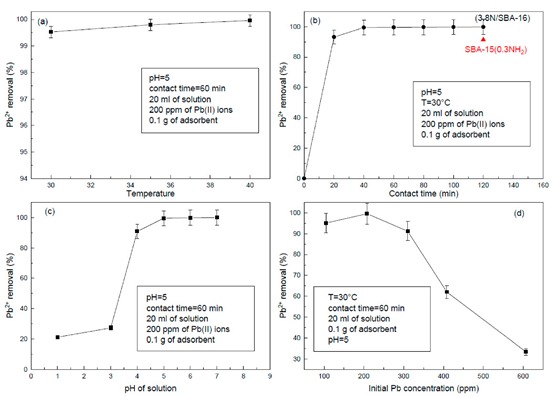
Influence of (**a**) temperature, (**b**) contact time, (**c**) pH, and (**d**) initial lead concentration on the Pb^2+^ removal from aqueous solution by the adsorption onto 3.8N/SBA-16. The SBA-15-based counterpart prepared with the same TEOS/APTES molar ratio of 3.3 is used as a reference (from [1]).

For the most optimized adsorbent 3.8N/SBA-16, a series of experiments were conducted to determine the optimum contact time, pH, adsorption temperature, and the initial lead concentration in the solution. The influence of temperature on the Pb^2+^ ions adsorption onto the 3.8N/SBA-16 sample is shown in Figure 9a. The adsorption was performed in the temperature range of 30–40 °C. As seen in this figure, the percentage of the Pb^2+^ removal in the temperature range studied was very high: 99.5% at 30 °C and 100% at 40 °C, suggesting that the adsorption process is endothermic. Because of the easy mobility of the molecules at 30 °C, this temperature was selected for further experiments. 

The effect of contact time on the adsorption capacity of the 3.8N/SBA-16 sample is shown in Figure 9b. The initial concentration of Pb^2+^ ions in aqueous solution was 200 mg L^−1^. As seen in Figure 9b, the initial rate of Pb^2+^ adsorption on the 3.8N/SBA-16 was fast, and equilibrium was reached after 40 min with total Pb^2+^ removal. Thus, the contact time of 60 min was chosen to perform further adsorption experiments. The comparison of the percentage of Pb^2+^ removal after a contact time of 120 min indicated a larger adsorption capacity of the 3.8N/SBA-16 sample with respect to its SBA-15-based counterpart [1] (99.8% vs. 91.5%). The fast adsorption of Pb^2+^ ions during the first 40 min strongly suggests the uniform distribution of-NH_2_ groups on the surface of SBA-16 adsorbent. 

The effect of variation of pH of solution on the capacity of Pb^2+^ adsorption on the most optimized adsorbent is shown in Figure 9c. In good agreement with a study on SBA-15-based adsorbent [1], the highest adsorption of Pb^2+^ was achieved at pH > 3. An increase of pH from 3 to 5 led to an increase of Pb^2+^ adsorption from 25.0 to 99.6wt.%. This result indicates that pH = 5 is the most appropriate to increase the extent of adsorption. This behavior may be likely related to the competitive adsorption of Pb^2+^, and H_3_O^+^ ions on the NH_2_-modified SBA-16 surface. At low pH, the number of H_3_O^+^ exceeds that of the Pb^2+^ ions being the surface of the adsorbent covered mainly by H_3_O^+^ ions leading to a lower extent of Pb^2+^ adsorption. When pH increases, more and more H_3_O^+^ ions leave the adsorbent’s surface, making the sites available to the Pb^2+^ adsorption [38]. 

Finally, the effect of initial lead concentration was evaluated (Figure 9d). Initial Pb^2+^ concentration was adjusted in the ranges of 100–600 mg L^−1^. As seen in Figure 9d, at a moderate initial lead concentration of 105 and 208 ppm, the adsorption of Pb^2+^ is high (95.1% and 99.5%, respectively). As expected, an increase in lead concentration from 200 to 600 ppm led to a drastic decrease in adsorption capacity. Assuming that the mechanism of lead adsorption on the siliceous adsorbents modified with -NH_2_ groups is probably through van der Waals electrostatic interaction of Pb^2+^ ions with surface -NH_2_ groups [1], at a higher initial lead concentration (>300 ppm), more Pb^2+^ was left in solution because lead in solution is much higher than the amount required to saturate the binding site. 

Summarizing, the SBA-16 functionalized with an optimized amount of aminopropyl groups demonstrated to be more efficient adsorbent than its SBA-15-based counterpart (Figure 8). The highest sorption capacity was achieved with the 3.3 TEOS/APTES molar ratio adsorbent due to its large specific surface area and unlimited accessibility for Pb^2+^ ions to -NH_2_ groups. The SBA-16-based adsorbents were stable until the temperature of 250 °C. The optimum pH value for removal of Pb^2+^ ions from aqueous solution was found to be in the range 5 to 6. The equilibrium for lead adsorption was reached at about 40 min. 

The comparison of the adsorption capacity of the 3.8N/SBA-16 studied in this work with that reported previously for the SBA-15-based counterpart [1] strongly suggests a larger adsorption capacity of the former with respect to the latter (39.8 and 36.4 mg of Pb^2+^ per g of adsorbent, respectively). The abilities of both adsorbents appeared to be closely related to their pore structure, pore density and the amount of the grafted amine groups. Indeed, because of the much larger amount of organoalkoxysilane precursor, the SBA-15 functionalized with (3-aminopropyl) trimethoxysilane exhibited a larger adsorption capacity [16] than SBA-15 functionalized with (3-aminopropyl) triethoxysilane (APTES) [1] (90 mg g^−1^ vs. 39.8 mg g^−1^). This could be explained in terms of the cage-like structure and two-times smaller pore diameter of SBA-16 with respect to SBA-15 counterpart. In such a case, additional Pb^2+^ ion trapping might well occur within the inner pore structure of SBA-16. Moreover, the easier accessibility of Pb^2+^ ions to amine groups as well as the steric difficulty for their leaving out from the cubic-structure smaller pores of SBA-16 adsorbent might explain the enhancement of its sorption capacity.

## 4. Conclusions

This work demonstrated that ordered SBA-16 mesoporous silica material grafted with an optimized amount of amine-functional groups is extremely effective in the removal of lead ions from aqueous solutions. 

The highest sorption capacity was achieved with the 3.3 TEOS/APTES molar ratio adsorbent. The cage-like structure of amine-modified SBA-16 demonstrated to be more effective for Pb^2+^ elimination from the aqueous solution than the two-dimensional channel system of amine-modified SBA-15. The amine-free SBA-16 adsorbent did not show Pb^2+^ ions adsorption at all. In the case of the SBA-16-based adsorbents, the easier accessibility of Pb^2+^ ions to amine groups as well as the steric difficulty for their leaving out from the cubic-structure smaller pores might explain the enhancement of its sorption capacity. Although this research was developed to study the adsorption capacity of the material to remove Pb (II) ions on ideal aqueous solutions, it is intended to prove its efficiency on real samples in future work, so the interference of more ions and organic materials could be assessed.

## Figures and Tables

**Table 1:** Some textural properties ^a^ of the amine-functionalized SBA-16 adsorbents and their adsorption efficiency for Pb^2+^ removal from aqueous solution ^b^.

Sample	N (mmol/g)	Pb/N (Atomic Ratio)	S_BET_ (m^2^/g)	Loss of S_BET_ (%)	V_total_ (cm^3^/g)	*d*_p_ (nm)
SBA-16	-	-	650	-	0.64	3.4
2.6N/SBA-16	2.6	0.07	507	22.0	0.45	3.1
3.8N/SBA-16	3.8	0.05	500	23.1	0.42	3.2
5.1N/SBA-16	5.1	0.03	494	24.0	0.43	3.1

^a^ As determined from N_2_ adsorption-desorption isotherms at 77 K; S_BET_: specific surface area calculated using Brunauer-Emmett-Teller (BET) equation; V_total_: adsorption total pore volume; dp: average pore diameter calculated from the isotherm adsorption branch.; ^b^ Adsorption efficiency expressed as Pb/N atomic ratio: adsorption conditions were: 0.1 of adsorbent, 20 mL of 200 mg L^−1^ of Pb^2+^ aqueous solution, pH = 5, contact time 60 min, T = 30 °C.

**Table 2:** Unit cell parameter (*a*_o_), and pore wall thickness (*w*_t_) of the bare SBA-16 and amine-functionalized adsorbents.

Sample	*a*_o_^b^ (nm)	*w*_t_^c^ (nm)	*w*_t_/*d*_p_ Ratio
SBA-16	14.13	8.84	2.6
2.6N/SBA-16	13.91	8.95	2.9
3.8N/SBA-16	13.91	8.85	2.8
5.1N/SBA-16	14.19	9.19	3.0

^b^ Calculated using the Equation (1); ^c^ Estimated using the Equation (2).

**Table 3:** Binding energies (eV) of core-electrons and surface atomic ratios of the most optimized 3.8N/SBA-16 adsorbent (from XPS) before and after Pb^2+^ ion adsorption.

XPS Data	Before Pb^2+^ Adsorption	After Pb^2+^ Adsorption ^a^
Si 2p (eV)	103.5	103.5
O 1s (eV)	532.7	532.7
N 1s (eV)	399.7 (60) 401.5 (40)	399.7 (63) 401.5 (37)
Pb4f_7/2_	-	138.8
N/Si	0.070	0.071 (0.109) ^b^
Pb/N	-	0.133 (0.139) ^b^

^a^ The adsorption conditions were: 0.1 g of adsorbent and 20 mL of aqueous solution containing an initial Pb^2+^ concentration of 200 mg L^−1^; pH = 5.0; contact time of 60 min; T = 30 °C; ^b^ Data of SBA-15(0.3NH_2_) sample (from [1]).

## References

[1] Morales V.H., Nava R., Silva Y.J.A., Sánchez S.A.M., Bueno J.L.P., Pawelec B. (2012). Adsorption of Lead (II) on SBA 15 Mesoporous Molecular Sieve Functionalized with -NH2 Groups. Microporoporous Mesoporous Mater..

[2] Vu D.-H., Bui H.-B., Bui X.-N., An-Nguyen D., Le Q.-T., Do N.-H., Nguyen H. (2019). A novel Approach in Adsorption of Heavy Metal Ions from Aqueous Solution Using Synthesized MCM-41 from Coal Bottom Ash. Int. J. Environ. Anal. Chem..

[3] Hong M., Yu L., Wang Y., Zhang J., Chen Z., Dong L., Zan Q., Li R. (2019). Heavy Metal Adsorption with Zeolites: The Role of Hierarchical Pore Architecture. Chem. Eng. J..

[4] Anirudhan T., Divya L., Ramachandran M. (2008). Mercury (II) Removal from Aqueous Solutions and Wastewaters Using a Novel Cation Exchanger Derived from Coconut Coir Pith and Its Recovery. J. Hazard. Mater..

[5] Sahoo J.K., Kumar A., Rout L., Rath J., Dash P., Sahoo H. (2018). An Investigation of Heavy Metal Adsorption by Hexa Dentate Ligand Modified Magnetic Nanocomposites. Sep. Sci. Technol..

[6] Mahmood-Ul-Hassan M., Suthar V., Ahmad R., Yousra M. (2018). Biosorption of Metal Ions on Lignocellulosic Materials: Batch and Continuous-Flow Process Studies. Environ. Monit. Assess..

[7] Swarnalatha K., Ayoob S. (2016). Adsorption Studies on Coir Pith for Heavy Metal Removal. Int. J. Sustain. Eng..

[8] Todorciuc T., Bulgariu L., Popa V. (2015). Adsorption of Cu (II) from Aqueous Solution on Wheat Straw Lignin: Equilibrium and Kinetic Studies. Cell Chem. Technol..

[9] Šćiban M., Klasnja M., Skrbic B. (2006). Modified Softwood Sawdust As Adsorbent of Heavy Metal Ions from Water. J. Hazard. Mater..

[10] Bernard E., Jimoh A. (2013). Adsorption of Pb, Fe, Cu, and Zn from Industrial Electroplating Wastewater by Orange Peel Activated Carbon. Int. J. Eng. Appl. Sci..

[11] Abdallah B., Baudu M., Derriche Z., Basly J. (2009). Aqueous Heavy Metals Removal on Amine-Functionalized Si-MCM-41 and Si-MCM-48. J. Hazard. Mater..

[12] Liu A.M., Hidajat K., Kawi S., Zhao D.Y. (2000). A New Class of Hybrid Mesoporous Materials with Functionalized Organic Monolayers for Selective Adsorption of Heavy Metal Ions. Chem. Commun..

[13] Kang T., Park Y., Yi J. (2004). Highly Selective Adsorption of Pt2+ and Pd2+ Using Thiol Functionalized Mesoporous Silica. Ind. Eng. Chem. Res..

[14] Nooney R.I., Kalyanaraman M., Kennedy G., Maginn E.J. (2001). Heavy Metal Remediation Using Functionalized Mesoporous Silicas with Controlled Macrostructure. Langmuir.

[15] Bruzzoniti M.C., Prelle A., Sarzanini C., Onida B., Fiorilli S., Garrone E. (2007). Retention of Heavy Metals Ion on SBA 15 Mesoporous Silica Functionalized with Carboxylic Groups. J. Sep. Sci..

[16] Aguado J., Arsuaga J.M., Arencibia A., Lindo M., Gascón V. (2009). Aqueous Heavy Metals Removal by Adsorption on Amine-Functionalized Mesoporous Silica. J. Hazard. Mater..

[17] Yang H., Xu R., Xue X., Li F., Li G. (2008). Hybrid Surfactant-Templated Mesoporous Silica Formed in Ethanol and Its Application for Heavy Metal Removal. J. Hazard. Mater..

[18] Giraldo L., Moreno-Piraján J.C. (2013). Study on the Adsorption of Heavy Metal Ions from Aqueous Solution on Modified SBA-15. Mater. Res..

[19] Da’Na E., Sayari A. (2012). Adsorption of Heavy Metals on Amine-Functionalized SBA-15 Prepared by Co-Condensation: Applications to Real Water Samples. Desalination.

[20] Cheraghali R., Tavakoli H., Sepehrian H. (2013). Preparation, Characterization and Lead Soption Performance of Aliginate SBA 15 Composite as a Novel Adsorbent. Scientia Iranica. F.

[21] Yang Y., Wang D. (2017). The Synthesis of Novel thiol/Amino Bifunctionalized SBA-15 and Application on the Cr (VI) Absorption. IOP Conf. Series: Earth Environ. Sci..

[22] Flodström K., Alfredsson V. (2003). Influence of the Block Length of Triblock Copolymers on the Formation of Mesoporous Silica. Microporous Mesoporous Mater..

[23] Huirache Acuña R., Pawelec B., Muñoz E., Nava R., Espino J., Fierro J.L.G. (2009). Comparison of the Morphology and HDS Activity of Ternary Co Mo W Catalysts Supported on P Modified SBA 15 and SBA 16 Substrates. Appl. Catal. B Environ..

[24] Brunauer S., Emmett P.H., Teller E. (1938). Adsorption of Gases in Multimolecular Layers. J. Am. Chem. Soc..

[25] Rouquerol J., Rouquerol F., Llewellyn P.L., Maurin G., Sing K. (2013). Adsorption by Powders and Porous Solids. Principles, Methodology and Applications.

[26] Grudzien R.M., Grabicka B.E., Jaroniec M. (2006). Adsorption and Structural Properties of Channel-Like and Cage-Like Organosilicas. Adsorption.

[27] Boss C.B., Fredeen K.J. (1989). Inductively Coupled Plasma Atomic Emission Spectrometry.

[28] Gallo J.M.R., Bisio C., Marchese L., Pastore H.O. (2008). Surface Acidity of Novel Mesostructurated Silicas with Framework Aluminium Obtained by SBA 16 Related Synthesis. Micropor. Mesopor. Mater..

[29] Sauer J., Marlow F., Schuth F. (2001). Simulation of Powder Diffraction Patterns of Modified Ordered Mesoporous Materials. Phys. Chem. Chem. Phys..

[30] Bottazzi G.S.B., Nartínez M.L., Costa M.B.G., Anunziata O.A., Beltramone A.R. (2011). Inhibition of the Hydrogenation of Tetralin by Nitrogen and Sulfur Compounds over Ir/SBA 16. Appl. Catal. A: Gen..

[31] Cheng C.-F., Lin Y.-C., Cheng H.-H., Chen Y.-C. (2003). The Effect and Model of Silica Concentrations on Physical Properties and Particle Sizes of Three-Dimensional SBA-16 Nanoporous Materials. Chem. Phys. Lett..

[32] Walcarius A., Etienne M., Bessiere J. (2002). Rate of Acess to the Binding Sites in Organically Modified Silicates. Amorphous Silica Gels Grafted with Amine or Thiol Groups. Chem. Mater..

[33] Ravikovitch P.I., Neimark A.V. (2002). Experimental Confirmation of Different Mechanisms of Evaporation from Ink-Bottle Type Pores: Equilibrium, Pore Blocking, and Cavitation. Langmuir.

[34] Zhao D., Huo Q., Feng J., Chmelka B.F., Stucky G.D. (1998). Nonionic Triblock and Star Diblock Copolymer and Oligomeric Surfactant Syntheses of Highly Ordered, Hydrothermally Stable, Mesoporous Silica Structures. J. Am. Chem. Soc..

[35] Boutros M., Onfroy T., Da Costa P. (2010). Mesostructured or Alumina-Mesostructured Silica SBA-16 As Potential Support for NOx Reduction and Ethanol Oxidation. Catal. Lett..

[36] Stevens W.J.J., Lebeau K., Mertens M., Van Tendeloo G., Cool P., VanSant E.F. (2006). Investigation of the Morphology of the Mesoporous SBA-16 and SBA-15 Materials. J. Phys. Chem. B.

[37] Pawelec B. (2004). HDS of Dibenzothiophene over Polyphosphates Supported on Mesoporous Silica. J. Catal..

[38] Decottiguies M., Phalippou J., Zarzycki J. (1978). Synthesis of Glasses by Hot Pressing of Gels. J. Mater. Sci..

[39] White L., Tripp C. (2000). Reaction of (3-Aminopropyl) dimethylethoxysilane with Amine Catalysts on Silica Surfaces. J. Colloid Interface Sci..

[40] Scott R.P.W. (1993). Silica Gel and Bonded Phases: Their Production, Properties & Use in LC.

[41] Chong A.S.M., Zhao X.S. (2003). Functionalization of SBA-15 With APTES and Characterization of Functionalized Materials. J. Phys. Chem. B.

[42] Luan Z., Fournier J.A., Wooten J.B., Miser D.E. (2005). Preparation and Characterization of (3 aminopropyl) triethoxysilane Modified Mesoporous SBA 15 Silica Molecular Sieves. Micropor. Mesopor. Mater..

[43] Payne J.C., Horst M.A., Godwin H.A. (1999). Lead Fingers: Pb2+ Binding to Structural Zinc Binding Domians Determined Directly by Monitoring Lead Thiolate Charge Transfer Bands. J. Am. Chem. Soc..

[44] Vögler A., Nikol H. (1992). Photochemistry and Photophysics of Coordination Compounds of the Main Group Metals. Pure Appl. Chem..

[45] Mariscal R., Soria J., Peña M.A., Fierro J.L.G. (1994). Structure and Reactivity of Undoped and Sodium Doped PbO/α Al2O3 Catalysts for Oxidative Coupling of Methane. Appl. Catal. A: Gen..

[46] Wagner C.D., Naumkin A.V., Vass A.K., Alisson J.W., Powell C.J., Rumble J.R.J. (2003). NIST X-Ray Photoelectron Spectroscopy Database 20 Version 3.4.

[47] García N., Benito E., Guzmán J., Tiemblo P., Morales V., Garcia R. (2007). Functionalization of SBA-15 by an Acid-Catalyzed Approach: A Surface Characterization Study. Microporous Mesoporous Mater..

